# Stereotactic Radiosurgery for Carcinoid Brain Metastasis: A Case Report

**DOI:** 10.7759/cureus.5509

**Published:** 2019-08-28

**Authors:** Felicia Cao, David M Sada, Syeling Lai, Yvonne H Sada

**Affiliations:** 1 Internal Medicine, Medical Scientist Training Program, Baylor College of Medicine, Houston, USA; 2 Radiology, Michael E. DeBakey Veterans Affairs Medical Center, Houston, USA; 3 Pathology, Michael E. DeBakey Veterans Affairs Medical Center, Houston, USA; 4 Hematology / Oncology, Baylor College of Medicine, Houston, USA

**Keywords:** carcinoid, brain metastasis, chemotherapy, temozolamide, rectal carcinoid, stereotactic radiosurgery

## Abstract

Carcinoid brain metastases are extremely rare and are associated with a poor prognosis. Treatment options are variable, ranging from surgery, radiation, or chemotherapy alone or combined. We report on a case of rectal carcinoid metastatic to the cerebellum and review chemotherapeutic regimens for carcinoid tumor treatment, focusing on the potential role of temozolomide or stereotactic radiosurgery.

## Introduction

Carcinoid tumors are rare, accounting for one to four cases per 100,000 population per year. They arise from neuroendocrine cells, with approximately 67% originating in the gastrointestinal system and 25% in the bronchopulmonary system. Though generally considered indolent lesions, carcinoid tumors can frequently metastasize, particularly those arising from the small intestine and colon. The most common sites for metastases are the regional lymph nodes, liver, lung, peritoneum, or pancreas [1]. Brain metastases are extremely rare, comprising 1.5% of cases, and are associated with a poor prognosis. The five-year survival for patients with carcinoid tumors with distant metastases excluding the brain is 40%, whereas the median survival for carcinoid tumor metastatic to the brain is reported to be less than a year [2]. We present a case of rectal carcinoid metastatic to the cerebellum and discuss treatment options for these rare tumors.

## Case presentation

A 59-year-old male presented to his primary care physician with a two- to three-week history of gait instability, tremor, and speech difficulty. He was initially diagnosed with carcinoid tumor of the rectum 18 years ago and received low anterior resection. Over the past seven years, he was found to have recurrent metastatic lesions to the lymph nodes, chest wall, liver, and pancreas. He had a prolonged treatment course, with multiple resections, including right hepatectomy of the liver lesion and chest wall excision, which had a Ki67 of 10%. The patient was previously given sunitinib and everolimus, but these were discontinued due to progression and medication intolerance, respectively. At the time of presentation, he was only on monthly octreotide 30 mg because he had declined further systemic therapy.

**Figure 1:**
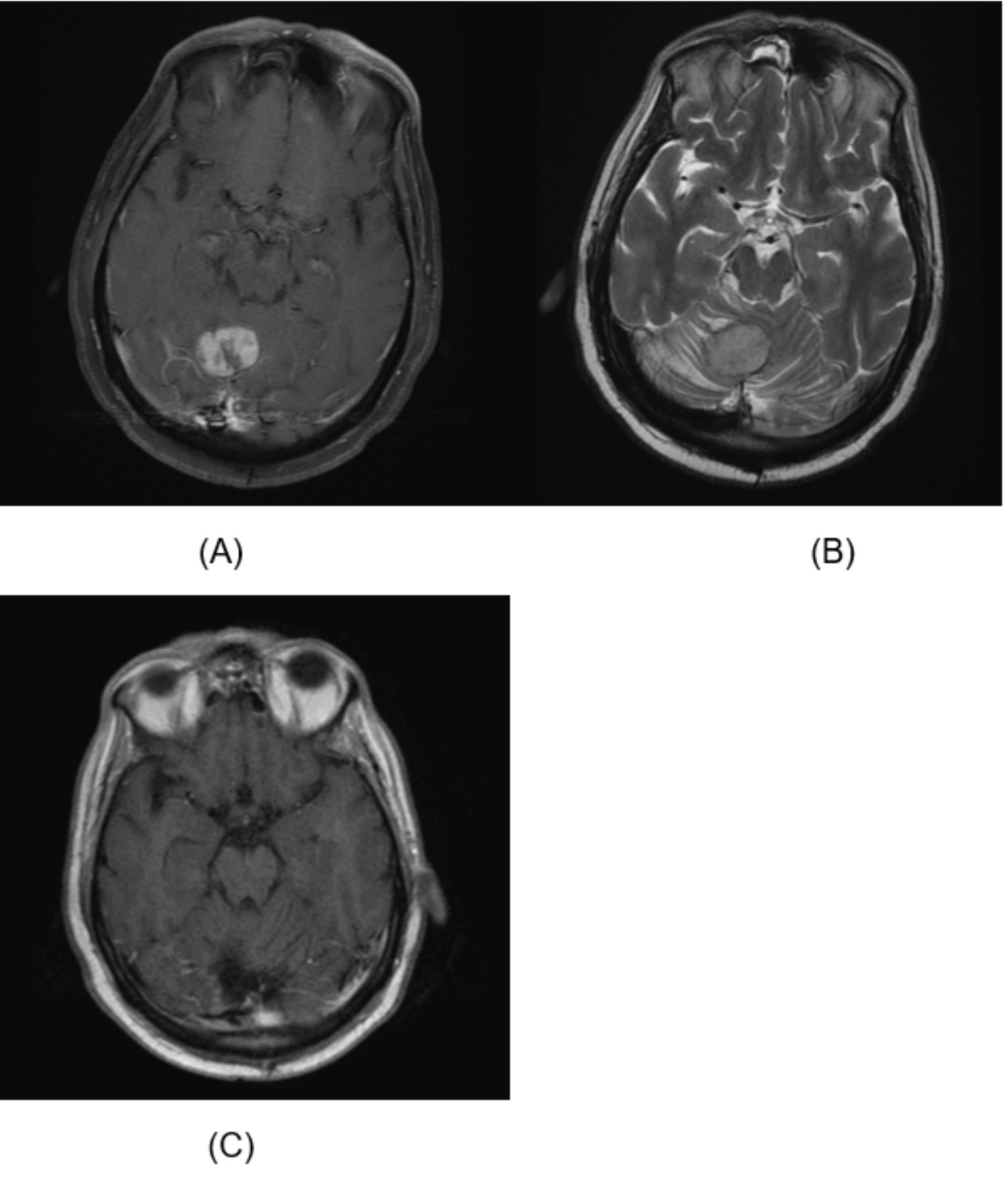
MRI of cerebellar mass (A) Axial T1 post-contrast image of the brain demonstrates an avidly enhancing cerebellar mass arising from the vermis. (B) Axial T2 MR image shows surrounding vasogenic edema and effacement of cerebellar sulci. (C) Post-surgical axial T1 post-contrast image of the brain demonstrates a surgical cavity with no evidence of residual neoplasm.

**Figure 2:**
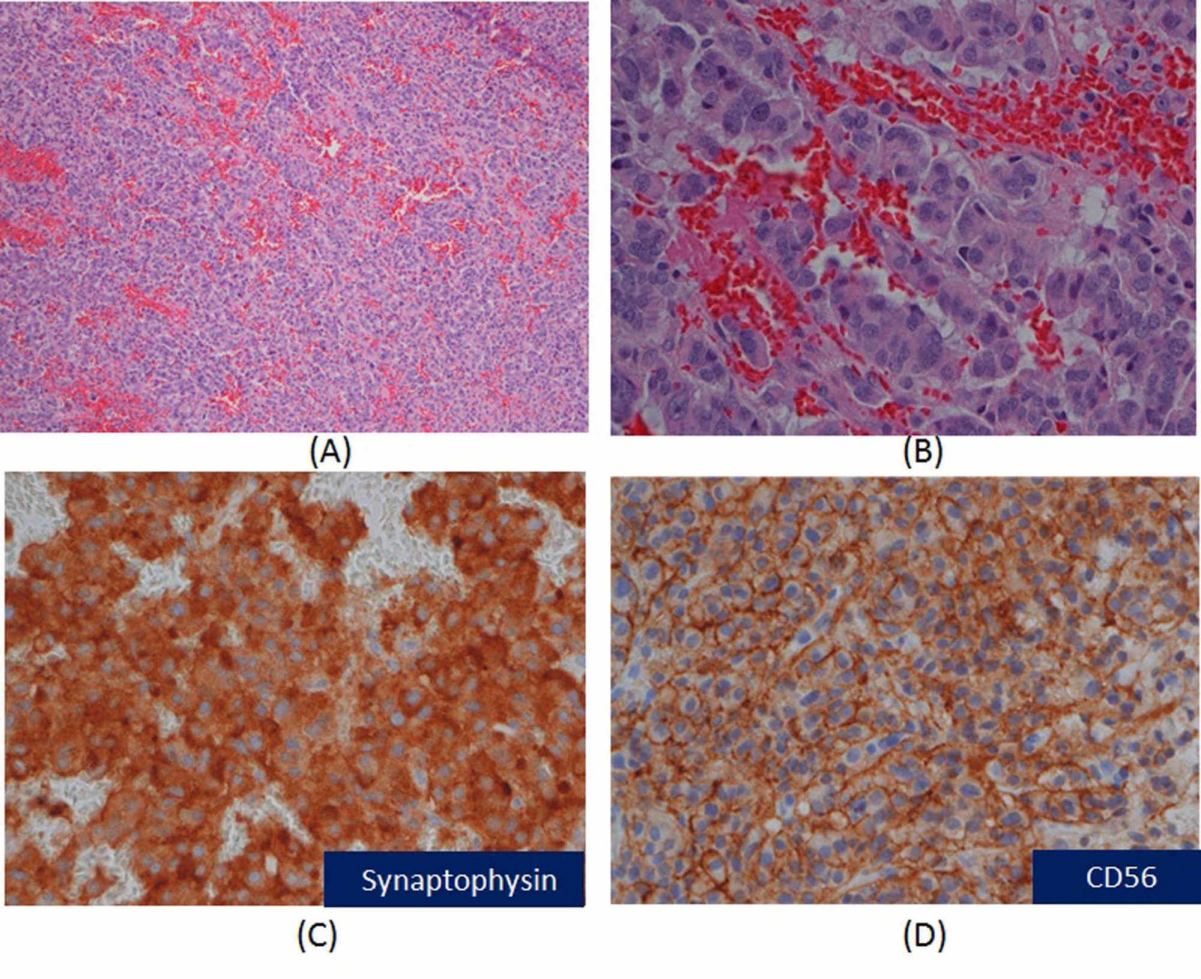
Microscopic findings of metastatic neuroendocrine tumor (A) Cellular organoid architecture with nests, cords, and glands in the background of rich vascular network, H&E stain, 100X. (B) Small to median tumor cells with eosinophilic and finely granular cytoplasm, uniform nuclei and finely stippled chromatin, H&E stain, 400X. (C) Immunostain for syneptophysin demonstrating diffuse cytoplasmic positivity in tumor cells, 400X. (D) CD56 stain showing strong cell membrane reactivity in tumor cells, 400X.

MRI revealed a three-centimeter mass in the cerebellar vermis with surrounding vasogenic edema (Figure 1). The mass was resected via suboccipital craniotomy, and pathology results revealed metastatic neuroendocrine carcinoma, with features similar to the prior chest wall resection and a Ki67 of 30% (Figure 2). His symptoms of gait instability, tremors, and speech difficulty resolved postoperatively. Patient received 2000 cGy in five fractions of stereotactic radiosurgery (SRS) to the cerebellar surgical bed, but elected against additional chemotherapy. Brain MRI performed five months after surgery shows no evidence of recurrence in the brain (Figure 1C). He remained symptom free and independent in his daily activities more than 11 months after resection of brain metastasis.

## Discussion

There are no definite treatment guidelines for intracranial metastasis from carcinoid tumors, due to the rarity of presentation. Treatment options include surgery, radiotherapy such as whole brain radiotherapy (WBRT) or SRS, or chemotherapy, either as monotherapy or in combination. The combination of WBRT and surgery has been shown to improve survival for carcinoid brain metastasis, with a median survival time of 3.2 years compared to 4.8 months for surgery alone and 6.0 months for WBRT alone [2]. Local radiation in the form of SRS has not been extensively studied in carcinoid brain metastasis due to the rarity of presentation. However, a phase III clinical trial evaluating postoperative SRS versus WBRT in resected solitary brain metastasis from multiple tumor types found no difference in overall survival between the two modalities [3]. Strikingly, WBRT was associated with more frequent decline in cognitive function than SRS, suggesting that SRS may be a less toxic adjuvant for the treatment of solitary brain metastasis.

The role of chemotherapy in the treatment of carcinoid brain metastasis is unclear. Previous case reports have shown that adjuvant chemotherapy with 5-ﬂuorouracil combined with irradiation of 45 Gy resulted in a survival of 12-18 months compared to adjuvant chemotherapy with 5-ﬂuorouracil and methotrexate, which resulted in a survival of nine months [4]. Somatostatin analogs, such as octreotide, are frequently used for systemic treatment of carcinoid tumors, since somatostatin receptors are expressed on the surface of carcinoid cells. However, somatostatin analogs have poor penetration into the blood-brain barrier, and thus are not an ideal choice for control of intracranial disease. Similarly, sunitinib and everolimus have poor penetration of the blood-brain barrier. In contrast, temozolomide, an oral cytotoxic alkylating agent, improves survival of patients with glioblastoma when combined with radiotherapy [5]. Temozolomide has also been used for the treatment of carcinoid tumors, though response rates are variable [6].

We conducted a review of temozolomide for the treatment of carcinoid tumors, summarizing a total of five trials (Table 1) [6-10]. Among the 21 carcinoid patients who received temozolomide as monotherapy, there were four partial responses and nine with stable disease, with a progression-free survival of only seven months [6]. Among 19 patients who received the combination of bevacizumab and temozolomide, there were no partial responses and 14 patients had stable disease, with a progression-free survival was only 7.3 months [7]. Best responses were seen among the 11 patients treated with the combination of capecitabine and temozolomide. There was one complete response, three partial responses, and three patients with stable disease, resulting in a progression-free survival of 10.5-14 months [8,9]. Lastly, among the 15 patients treated with the combination of temozolomide and thalidomide, there was one partial response and a median response duration of 13.5 months [10]. Unfortunately, none of the trials included patients with carcinoid brain metastasis.

**Table 1:** Summary of Temozolomide Regimens for Treatment of Carcinoid Tumors PR: Partial response SD: Stable disease CR: Complete response PFS: Progression-free survival

Regimen	Number of Carcinoid Patients	Number of Total Patients	Prior Therapy	Response	PFS (Carcinoid Only)	PFS (All Patients)	Reference
Bevacizumab 5 mg/kg D1, 15; temozolomide 150 mg/m^2^ D1-7, 15-21	19 carcinoid (1 appendix, 7 small bowel, 4 bronchial, 7 unknown primary)	34	Included octreotide, embolization, chemotherapy, sunitinib, radiofrequency ablation, interferon, or radiation	0/19 PR, 14/19 SD	7.3 months	11 months	[7]
Capecitabine 600 mg/m^2^ D1-14; temozolomide 150 mg/m^2^ D10-14	7 carcinoid (1 stomach, 2 ileum, 1 right colon, 3 rectum)	21	Progression after failure on long-acting sandostatin analog, first-line chemotherapy with platinum + VP16	2/7 PR, 2/7 SD	10.5 months	16.5 months	[8]
Capecitabine 600 mg/m^2^ D1-14; temozolomide 150-200 mg/m^2^ D10-14	4 carcinoid (2 foregut, 2 midgut)	18	Progression on 60 mg/month octreotide chemotherapy, or chemoembolization	1/4 CR, 1/4 PR, 1/4 SD	14 months	14 months	[9]
Temozolomide 100-200 mg/m^2^ for 5 consecutive days	21 carcinoid (1 gastric, 7 thymic, 13 bronchial)	36	Previously had received a mean of 2.4 systemic antitumor regimens	4/21 PR, 9/21 SD	7 months	7 months	[6]
Temozolomide 150 mg/m^2^ D1-7, 15-21; thalidomide 50-400 mg daily	15 metastatic carcinoid	28	Chemoembolization, octreotide, chemotherapy (platinum-based)	1/15 PR	Not reached, median response duration 13.5 months	Not reached, median response duration 13.5 months	[10]

Peptide receptor radioligand therapy (PRRT) using 177Lu-dotatate has emerged as a novel approach to treating neuroendocrine tumors with Ki67 ≤ 20% that demonstrate somatostatin receptor expression on imaging and have progressed on a somatostatin analog. The randomized phase III NETTER-1 trial reported an improved progression-free survival of 20 months and higher response rate using PRRT compared to placebo. Brain metastasis was not reported as a metastatic site in the trial, and the effect of PRRT on overall survival is unknown [11]. However, one case report has demonstrated the use of PRRT in a patient with a pituitary metastasis from an ileal neuroendocrine tumor; the patient had a partial response and tolerated treatment well [12]. This suggests that PRRT can also cross the blood-brain barrier and may be considered as a possible therapeutic option for patients with rare carcinoid brain metastasis. 

## Conclusions

We presented a case of rectal carcinoid metastatic to the brain that responded to surgery and SRS. Though brain metastasis are rare, patients with a history of carcinoid tumor presenting with new neurological symptoms must be evaluated with imaging to rule out potential metastatic disease. Only WBRT has been shown to improve survival for carcinoid brain metastasis. However, SRS may be a newer modality for the treatment of solitary carcinoid brain metastasis with similar efficacy but reduced toxicity. The evidence for adjuvant chemotherapy to treat brain carcinoid metastasis is limited, but the use of temozolamide should be considered. The combination of capecitabine with temozolomide appears to be superior to bevacizumab with temozolomide, thalidomide with temozolomide, or temozolomide monotherapy. PRRT may also be a well-tolerated treatment approach for carcinoid brain metastasis.
